# Ocular and Superficial Body Thermographic Findings in Sled Dogs before and after Competition

**DOI:** 10.3390/ani13050854

**Published:** 2023-02-26

**Authors:** Giuseppe Spinella, Andrea Galimberti, Giorgia Casagrande, Sergio Maffi, Vincenzo Musella, Simona Valentini

**Affiliations:** 1 Department of Veterinary Medical Sciences, University of Bologna, Ozzano dell’Emilia, 40064 Bologna, Italy; 2 SVA Taddeo & Galimberti, U/O Clinica Veterinaria Caravaggio—Circ. Specchio 20/22, 24043 Caravaggio, Italy; 3 Clinica Veterinaria Maffi, Piazza Vincenzo Rosa, 10, 25036 Palazzolo sull’Oglio, Italy; 4 Department of Health Sciences, University Magna Graecia, 88100 Catanzaro, Italy

**Keywords:** sled-dog, thermography, Siberian Husky, sled, dog

## Abstract

**Simple Summary:**

Thermography may provide useful data during competitions with sled dogs for rapid clinical screening during activity. The results of this study showed a significant increase in post-competition ocular temperature of both eyes, regardless of the length of the race. Superficial body temperatures also increased after competition, but it was particularly affected by environmental and subjective factors.

**Abstract:**

Competitions involving sled dogs are rapidly growing and body temperature assessment could represent a prompt and non-invasive method of screening for potential pathological conditions during or after activity. The aim of this clinical study was to evaluate if thermography is able to monitor the pre- and post-competition ocular and superficial body temperature variations during a sled dog competition. It subsequently compared the data relating to the ocular temperatures in different race types: mid-distance (30 km) and sprint (≤16 km). Results showed a statistically significant increase in post-competition ocular temperature of both eyes, regardless of the length of the race. The relative increase in the temperatures of the other body surfaces was lower than the expected values, probably due to the influence of environmental and subjective factors such as the type of coat of the Siberian Husky or subcutaneous fat. Infrared thermography has therefore proved to be useful method in sled dog competition conditions for screening superficial temperature variations, as the investigation is normally conducted in an external environment and often in demanding work conditions.

## 1. Introduction

Research on the history of the sled dog suggests that the use of the dog as a draft animal appears to date back to at least 8000 to 9000 years ago and it reached a particularly high level among the nomadic tribes of Siberia (Chukchi and Samoyed) [1,2,3]. 

Today, the sled dog is limited to tourist, recreational or competitive activities; in particular, there are three main sports disciplines: sleddog, pulka and skijoring. Races vary enormously from long-distance racing (from 250 to 1600 km in length), such as the well-known Iditarod in Alaska or the Finnmarksløpet and the Femundløpet in Norway, to mid-distance racing (30–250 kms) or sprint racing (generally 4–20 km).

Recently, the athlete role of canines has seen great improvement in terms of media and scientific involvement, with an increase in knowledge of the physiological requirements of sled dogs, promoting and encouraging physical well-being and veterinary clinical health surveillance during competitive events [4]. However, although a standard official protocol to monitor sport dog fitness has not yet been approved, it is usual practice for veterinarians working in dog sled competitions to perform microchip recognition and a pre-start clinical evaluation. Environmental parameters in sledding competition contexts limit the use of many diagnostic tools, either portable or non-invasive instruments, which could allow immediate and reliable results and help diagnostic screening during clinical examinations in the field. The assessment of body temperature in the sled dog represents a non-invasive monitoring method of canine thermoregulation during activity, consequently acquiring a value of non-invasive clinical screening of potential pathological conditions.

During intense physical activity, the energy produced by muscular effort is generally converted into metabolic heat: in the dog, heat is then dissipated through the respiratory system, evaporation processes [5], salivation and painting [6], resulting in water loss that could lead to signs of dehydration and increased body temperature [7,8,9].

In competitive and environmental contexts such as those reported for sled dogs, the rectal temperature measurement, generally performed in clinical trials, necessarily requires canine containment and can be extremely uncomfortable for the dog. 

The thermographic technique was used for the first time in equine medicine by Smith [10] and it is currently widely used especially for the diagnosis and follow-up of pathologies involving the locomotor system [11]. Additionally, in the sporting context, the use of a thermal imaging camera is a valid aid during equine training for monitoring muscle warm-up and verifying the presence of asymmetries in thermal distribution [12,13]. 

Thermography has also recently been applied in canine sports training and could represent a non-invasive screening tool for pathological conditions, such as potential injuries to the musculoskeletal system [4] or stress diseases that will then require further diagnostic investigations [14,15,16]. However, to the best of the authors’ knowledge, no specific publications have been produced on thermography application in sled dogs. 

The aims of this study were to: 1. evaluate thermography as a screening tool to monitor the potential variation of the pre-and post-race superficial body and ocular temperatures in sled dogs; 2. compare the ocular temperatures obtained in different competitions (mid-distance race and sprint race, of 30 km and ≤16 km of length, respectively, according to Italian national regulations).

## 2. Materials and Methods

Ethical approval for this study was obtained by the Ethical Committee of the University of Bologna (protocol number: ID 914/2018). 

Thirty-three Siberian Huskies (10 intact females, 2 spayed females and 21 intact males), mean age 63.9 ± 27.08 months (median 65 months), mean weight 21.27 ± 3.59 kg (median 20 kg) and Body Condition Score between 3.5 and 5.5 (following guidelines for Body Condition Score of International Sled Dog Veterinary Medical Association), attending the First Cortina International trial sporting event (Italy) on 26–27 February 2021, were included in this study. Fourteen dogs attended the mid-distance competition and nineteen dogs the sprint races on tracks equal to or less than 16 km. 

Soundness status was routinely evaluated before the competition by a licensed doctor in veterinary medicine.

Thermographic images were taken using a portable thermo camera, FLIR T540 24° (T540, FLIR Systems Inc., Danderyd, Sweden), with infrared (IR) resolution 464 × 348 pixels. The field of view was 53° × 41°. Images were recorded and analysed with FLIR Tools software (FLIR Systems Inc., Danderyd, Sweden).

The investigation was carried out in the stakeout of each team. Pre-competition thermographic scans (T0) were acquired up to two hours before the start, compatibly with the arrival of the mushers in the Cortina. The post-competition images (T1) were taken no later than 20 min after the end of the race from 10:30 to 12:00. The thermal images were taken out of direct sunlight and wind after removing the harness immediately after the race. The camera was handheld at approximately 1 metre away from the dog head (ocular measurements) and 1.5 m up from other regions in order to include all the regions of interest (ROIs). Images were taken on dogs tied into the stakeout or kept on a leash by the musher in standing position. Generally, the musher was allowed to support the dog’s head to measure eye temperatures.

A total of six thermographic images were acquired for each dog: a. face region; b. forelimb cranial view; c. right lateral body; d. left lateral body; e. hindlimb caudal view; f. lumbo-sacral region dorsal view. 

For each thermal image, one or more ROIs have been identified, for a total of 15 ROIs (Table 1).

**Figure 1:**
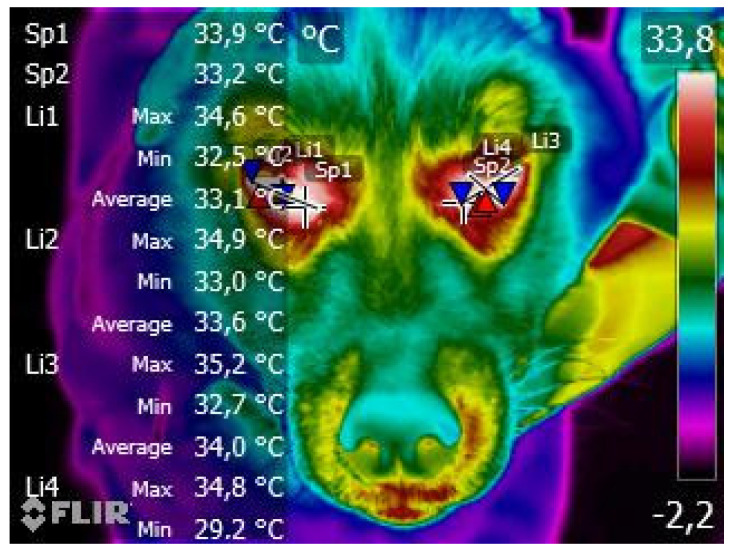
Five-and-a-half-year-old, female, Siberian Husky dog. Example of ocular measurement as the average value resulting from the three different values obtained on the hotspot centered on the medial canthus of the eye and on two perpendicular lines, drawn from the dorsal to the ventral margin of the eye and from the ocular medial canthus to the lateral one. The brighter colors (red, orange, and yellow) indicate warmer temperatures (more heat and infrared radiation emitted), while the purples and dark blue/black indicate cooler temperatures (less heat and infrared radiation emitted). Moreover, all commas inserted in the measurements within the figure are indicated according to the Italian style, therefore they must be understood as points to indicate decimals following the British style (e.g., “33,9” in Italian style has to be intended as “33.9” following the British style). Sp = Spot. Li = Line. Regarding the other ROIs, elliptical areas corresponding to the main body regions were identified within each image, as well as lines (major axis of the ellipse) and points within the areas: the final surface temperature was obtained from the average of the measurements obtained in areas, lines and points (Figure 2).

The ocular temperature was evaluated as the average value resulting from the three different values obtained: the first one, by the hotspot centred on the medial canthus of the eye and the second and third ones by the mean value of two perpendicular lines, drawn from the dorsal to the ventral margin of the eye and from the medial ocular canthus to the lateral one (Figure 1).

All data were submitted to descriptive (mean ± standard deviation-SD and median) and analytic statistical analysis.

A paired T-test was applied to investigate differences between the pre- and post-race temperature in the different ROIs. Dogs were also divided into two groups according to the distance of the race (mid-distance (30 km) and sprint (≤16 km) and the Mann–Whitney U test was used to compare the difference in ocular temperatures at the end of the race (T1). The significance level was set for a *p* value ≤ 0.05. All data were analysed using Stata version 16 software (StataCorp, College Station, TX, USA-2019).

## 3. Results

In total, 66 ocular thermographic images and 429 superficial body images were analysed. The environmental temperature ranged from −5 °C to +3 °C and from 8 °C to 10 °C when data at T0 and T1 were collected, respectively.

**Figure 3:**
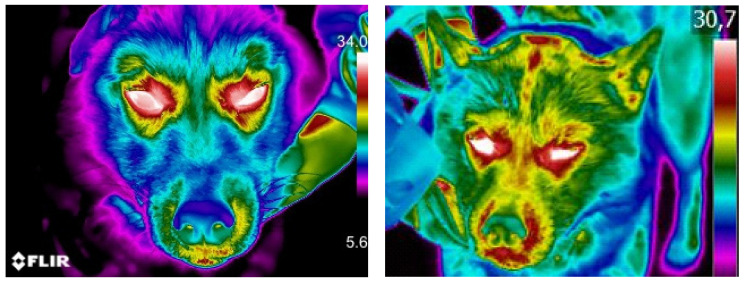
Five-year-old, female, Siberian Husky dog. Comparison of macroscopic thermographic aspect of ocular image before (T0) and after competition (T1). The brighter colors (red, orange, and yellow) indicate warmer temperatures (more heat and infrared radiation emitted), while the purples and dark blue/black indicate cooler temperatures (less heat and infrared radiation emitted).

Considering the entire group of dogs, in the pre-competition phase (T0) the mean temperature of the right eye was 32.98 ± 1.68 °C, while in the left eye the mean value was 32.83 ± 1.57 °C; in the post-race (T1) the mean temperature was 35.08 ± 0.95 °C and 34.95 ± 0.88 °C for the right and left eye, respectively (Table 2 and Figure 3).

When comparing the mid-distance (30 km) and sprint (≤16 km) groups of dogs, the ocular temperature mean value at T0 in the mid-distance group was 32.49 ± 1.34 °C in the right eye and 32.17 ± 1.37 °C in the left eye; the ocular temperature mean value at T0 in the sprint group was 33.34 ± 1.84 °C in the right eye and 33.32 ± 1.56 °C in the left eye. The ocular temperature mean value at T1 in the mid-distance group was 35.24 ± 0.96 °C in the right eye and 35.24 ± 0.56 °C in the left eye; the ocular temperature mean value at T1 in the sprint group was 34.97 ± 0.95 °C in the right eye and 34.74 ± 1.01 °C in the left eye (Table 3).

The statistical analysis showed a significant increase in ocular temperature in all dogs examined (N = 33) between T0 and T1 for both the right and left eyes (*p* < 0.0001).

No significant difference in the post-race ocular temperature between the mid-distance (30 km) and the sprint category group (≤16 km) were observed for either eye, right (*p* = 0.49) or left (*p* = 0.12).

**Figure 4:**
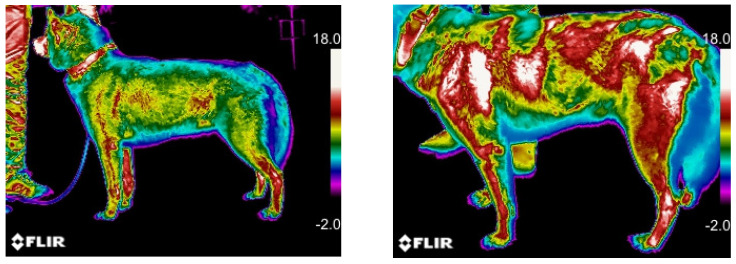
Five-year-old, female, Siberian Husky dog. Comparison of macroscopic thermographic aspect of left lateral view of the body surface before (T0) and after competition (T1). The brighter colors (red, orange, and yellow) indicate warmer temperatures (more heat and infrared radiation emitted), while the purples and dark blue/black indicate cooler temperatures (less heat and infrared radiation emitted). -2.0 = −2.0.

The mean ± SD and median values of body surface temperature in the different ROIs are reported in Table 4. A statistically significant increase in the temperature of the body surfaces of all ROIs between T0 and T1 was recorded (*p* < 0.05) (Figure 4).

## 4. Discussion

The aims of this study were to investigate if thermography could be used as a screening technique to (1) monitor the variation pre- and post-competition in superficial body temperature and ocular temperatures and (2) evaluate if ocular temperatures changed in different distance competitions.

The results showed that thermography was able to monitor the increase in body temperatures secondary to physical activity: a statistically significant increase in superficial and ocular temperatures was reported, but no significant differences were detected for ocular temperatures when mid-distance and sprint competitions were compared.

Body temperature variations during exercise have been widely documented in domestic mammals, as an effect of the increase in muscle metabolism with changes in respiratory and heart rates [18,19], and thermal loss during exercise may alter normal homeostasis. Changes in body temperature, as an index of the physiological response to exercise, has to therefore be correctly monitored; however, it should be performed without generating stress in the dog, especially in a competitive context. For this reason, thermography could represent a suitable non-invasive tool for screening. The use of thermography in the context of sled dogs is not yet widespread, even if this technique has generally been used during clinical examinations of dogs and for research aims in *La Grande Odysseée* [20].

In our experience, during sled dog competitions, the infrared camera for temperature measurement was easy to manage and particularly helpful, as it is a non-invasive portable screening tool that allows quick screening of superficial temperature changes. Image acquisition often occurs in challenging working conditions for climatic and organisational reasons; consequently, the T0 acquisition was often determined by the availability of the mushers for the dog’s examination, while at T1 the main difficulty was related to the simultaneous arrival of different teams participating in our study.

This standard protocol resulted in a limited increase (5–10 min) in the whole clinical examination, routinely scheduled before the competition. However, the thermographic evaluation was carried out before the physical examination in order to avoid any clinical manipulation, especially regarding the musculoskeletal system, which could generate artefacts.

The distance between camera and patient was set at 1 metre for ocular images and 1.5 m for other ROIs.

Although most publications on thermography for clinical purposes describe distances less than [14,21] or equal to 1 metre [16,22] from the target region, in accordance with the guidelines prepared by the American Academy of Thermology, which report an ideal distance from the subject of 3–8 feet (0.9–2.4 m), we have standardised a distance to 1 and 1.5 m to allow us to correctly include in the frame (wide-angle photographic lens) all ROIs that would then be used within each specific projection. However, other studies on dogs have evaluated body surface temperatures with a dog–camera distance varying between 0.3 and 0.67 m [16,23,24].

The operator who acquired the images was a well-trained and experienced practitioner. Vainionpää and collaborators (2012) reported that, although the thermographic technique does not depend on the operator who performs the examination or interprets it, it is fundamental that the operator is adequately trained to reduce the differences related to the methods of acquisition and interpretation of thermal images [25].

The medial canthus of the eye has been reported as an anatomical point with the highest temperature on the eye, also compared to the lateral canthus [14]. Other studies have measured the ocular temperature within a rectangular area that included the ocular globe and about 1 cm of the eyelids [22] or only at the level of the lacrimal caruncle [16]. We decided to apply a measurement technique similar to that described by Elias, which, in our opinion, was easier to perform in the field [14]. The applied technique allowed the examination of several points of the eye surface in order to perform a wide mapping of the ocular temperature.

Thermal images to measure body surface temperature were acquired on standing dogs obtaining five projections: front limbs in cranial view, right lateral body projection, left lateral body projection, hind limbs in caudal view and dorsal view of the lumbosacral region. These five projections made it possible to analyse 13 ROIs. Other studies have identified in the thermal images the areas centred on the main anatomical regions, standardised according to the width of the main muscle groups (neck, shoulder, arm, back, rib, rump and thigh) [26] or by using specific sub-regions centred around specific muscle groups [24]. We decided to identify the ROIs as generic anatomical areas, as our aim was to confirm by thermography the systemic superficial heating of the dogs (body and eyes) after races and not a specific involvement of individual muscle groups during activity.

Our data confirmed a statistically significant increase in ocular and body temperature after physical activity, regardless of the distance of the race; an evident uniformity of ocular temperatures resulted between the pre- and post-race values, while larger standard deviations of the means of the superficial body temperatures were observed, as they probably were more affected by environmental factors.

The values of the eye temperatures collected in the pre- and post-race outdoor environment were found to be similar to those measured in indoor conditions with controlled temperature and humidity on dogs that exercised on a treadmill [16]. Rizzo and collaborators reported that the mean value for both eyes was 33.29 ± 1.6 °C at rest and 35.49 ± 0.52 °C after 10 min of treadmill exercise [16].

In our study, the comparison between the two different typologies of races revealed no difference at T1, leading us to conclude that ocular temperature increases regardless of the distance travelled during the run. These data were in line with results of other previous thermographic studies, which had shown that ocular temperature increases uniformly during exercise [14,16], as well as results described by Phillips et al. (1981) relative to rectal temperature in running sled dogs [9]. Moreover, ocular temperature between the pre- and post-race increased by 2.1 °C on average, in line with that reported by Rizzo and collaborators [16]. In contrast, Zanghi (2016) observed differences in post-exercise eye temperature increase depending on breeds [22]. For practical reasons due to competition rules, the ocular temperature was not compared with the rectal temperature, which would have allowed an effective monitoring of changes in the internal body temperature [22]; however, measurement of rectal temperature is not part of the routine pre-race examination.

With particular attention to superficial body temperature, our results showed a significant increase post-competition in all examined ROIs.

However, pre-race superficial body temperatures were lower in the CDDX ROIs (mean and standard deviation 5.11 ± 2.78 °C), CDSX (5.29 ± 2.82 °C) and LGS (4.99 ± 3.34 °C). It has been speculated that the body surface temperature of mammals is about 5 ° C lower than rectal temperature [27], but our results significantly deviate from the expected values. The thickness and type of coat of the Siberian Husky may have influenced the measurement of the body surface temperature, limiting a real surface measurement, worsened by the presence of snow or sleet on the coat. The coat of the Siberian Husky is composed of a dense undercoat slightly topped by guard hairs. These characteristics allow the interposition of an insulating layer of air between the skin and the hair, limiting the propagation of thermal radiation [20]. The preparation of the dogs, according to the protocol for thermographic examination performed in a closed and controlled environment by Soroko and collaborators (2021), involves combing the hair one hour before the exercise session [26]: this practice would make it possible to flatten the hair and limit the isolation layer of air. However, this manipulation was not feasible in our context.

Relative to the higher temperature in the ROIs of the right anatomical regions (ODX, LDX, CDX and GDX) observed at T1, we hypothesise that it could be due to the sun position that for most of the race radiated the right side of the dogs.

Environmental parameters may affect thermal detection during physical activity: significant association was observed between the environmental and body temperatures in sled dogs competing between −9 and 25 °C [9]. Philips and collaborators showed that sled dogs are mostly affected by mid–high ambient temperatures (≥15 °C), with an average body temperature increase of up to 4.6 °C, in contrast to the 2 °C mean increase when ambient temperatures were <15 °C [9]. Sled dogs tolerate environmental temperatures below 0 °C well, as these dogs spend a lot of time and compete outdoors [28]. Nevertheless, in our study it was not possible to exclude that the range of environmental temperature variation between 0 and 10 °C during the race did not partially influence the final evaluations.

Relative to ocular temperature, Zanghi B.M. (2016) observed a return to the physiological value within 30 min indoors in Beagle dogs, unlike Labrador Retrievers. Differently, rectal temperature returned to physiological values in 15 min in Beagles and in 30 min in Labrador Retrievers [22]. Another study conducted by Diverio and collaborators (2016) monitored rectal temperature in avalanche dogs: these authors reported activity-induced rectal temperature changes that returned to the normal range two hours after the end of the research activity [29]. Similar results on working dogs were also observed by Spinella and collaborators (2021) [30].

The main limitation of this study concerned the acquisition of thermal images outdoors, as it was not possible to recreate standard environmental conditions to limit the influence of external factors such as ambient temperature, snow-covered ground and sunlight, which could cause artefacts. However, our aim was to obtain information in real race conditions.

Only Siberian Husky dogs were included in this study, because this breed is consolidated from an ethnographic and health point of view and, therefore, guarantees a more homogeneous sample [31]. The Husky’s undercoat provides excellent thermal insulation and may give a biased result, but on the other hand all the breeds used in this specific activity have similar haircoat features.

Moreover, as previously reported, the anticipation of activity could be associated with an increase in rectal temperature in pre-race timing [18], but this does not appear to have affected the thermography’s ability to determine the temperature changes before and after the race.

Furthermore, the dogs examined at the stakeouts were sometimes initially lying in the snow: in the literature, it is reported that ice on the fur surface could affect thermal images, particularly when it melts, as the humidity could increase surface evaporation [25].

Finally, the number of included dogs (33 dogs) represented only 7.5% of the total sample that took part in the competition; although a good number of dogs were examined, these were not equally distributed between the races.

## 5. Conclusions

Thermography is a useful method during the initial screening and in the follow-up during clinical visits in sled dog sports competitions, especially in the pre-competition, where it is advisable to minimize stress.

Ocular temperature was found to be more reliable than the temperature of the other superficial regions. Our data confirmed that thermography can detect the increase in post-race ocular temperature, which was not correlated to the distance travelled by the dogs.

Thermographic evaluations of other superficial areas also showed an increase in post-competition temperatures secondary to physical exertion. However, this increase was less than the expected values, probably highlighting the limit represented by individual and environmental factors.

## Figures and Tables

**Figure 2:**
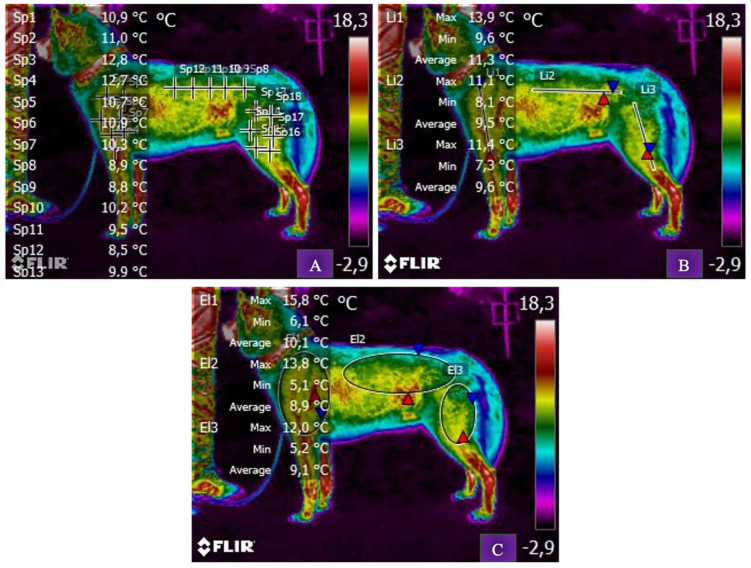
Five-and-a-half-year-old, female, Siberian Husky dog. Pattern of measurement of left homobrachial region (OSX), thoraco-lumbar region and left dorsal portion of the ventro-lateral region of the abdomen (LSX) and left thigh region (CSX). For each ROI, the body surface temperature was measured using three techniques: by points of interest (**A**), by lines (**B**) and by areas (**C**). The brighter colors (red, orange, and yellow) indicate warmer temperatures (more heat and infrared radiation emitted), while the purples and dark blue/black indicate cooler temperatures (less heat and infrared radiation emitted). Moreover, all commas inserted in the measurements within the figure are indicated according to the Italian style, therefore they must be understood as points to indicate decimals following the British style. Sp = Spot. Li = Line. El = elliptical area.

**Table 1:** Description of the fifteen ROI taken into consideration in the examination and relative anatomical regions as reported by Shaller in Illustrated Veterinary Anatomical Nomenclature in 1992 [17].

	Regions of Interest (ROIs)	Anatomical Regions
1	Right eye	
2	Left eye	
3	Right pectoral subregion of the sternal region (PDX)	Presternal region
4	Left pectoral subregion of the sternal region (PSX)	Presternal region
5	Right homobrachial region (ODX)	Scapular region, region of scapular cartilage, supraspinatus region, infraspinatus region, shoulder joint region
6	Left homobrachial region (OSX)	Scapular region, region of scapular cartilage, supraspinatus region, infraspinatus region, shoulder joint region
7	Right lateral view of the thoraco-lumbar region and right dorsal portion of the ventro-lateral region of the abdomen (LDX)	Region of the thoracic vertebrae, lumbar region, paralumar fossa
8	Left lateral view of the thoraco-lumbar region and left dorsal portion of the ventro-lateral region of the abdomen (LSX)	Region of the thoracic vertebrae, lumbar region, paralumar fossa
9	Lateral view of the right thigh region (TDX)	Region of thigh
10	Lateral view of the left thigh region (TSX)	Region of thigh
11	Lateral-caudal view of the right leg region (LeDX)	Crural region, region of common calcaneal tendon, popliteal region
12	Lateral-caudal view of the left leg region (LeSX)	Crural region, region of common calcaneal tendon, popliteal region
13	Caudal view of the thigh and right leg region (TLDX)	Region of thigh, crural region, region of common calcaneal tendon, popliteal region
14	Caudal view of the region of the thigh and left leg (TLSX)	Region of thigh, crural region, region of common calcaneal tendon, popliteal region
15	Caudal view of the lumbar region, sacral region and dorsal portion of the hip or gluteal region (LSG)	Lumbar region, sacral region, region of the root of the tail, gluteal region, *regio clunis*

**Table 2:** Eye temperature: mean value ± SD and median obtained in T0 and T1 in the entire group. All values are expressed in °C. Data in T1 were significantly increased (*).

*(ROI)*	*T0*		*T1*	
	Mean Value ± SD	Median	Mean Value ± SD	Median
*RIGHT EYE*	32.98 ± 1.68	33.20	35.08 ± 0.95 *	34.90
*LEFT EYE*	32.83 ± 1.57	33.00	34.95 ± 0.87 *	35.00

**Table 3:** The table summarizes the mean ± SD and median temperature values of the right and left eye in T0 and T1 in dogs that participated in the mid-distance and sprint competition. All values are expressed in °C. No significant differences were observed between T1 values comparing mid-distance and sprint races.

	*Right Eye*		*Left Eye*	
*T0*	Mean Value ± SD	Median	Mean Value ± SD	Median
*Mid-distance*	32.49 ± 1.34	31.90	32.17 ± 1.37	31.85
*Sprint*	33.34 ± 1.84	34.10	33.32 ± 1.56	33.80
*T1*				
*Mid-distance*	35.24 ± 0.96	34.95	35.24 ± 0.56	35.30
*Sprint*	34.97 ± 0.95	34.90	34.74 ± 1.01	34.70

**Table 4:** Mean ± SD and median temperature values of the ROIs in T0 and T1.

	*T0*		*T1*	
*ROI*	Mean ± DS	Median	Mean ± DS	Median
*PDX*	6.02 ± 3.33	5.90	14.41 ± 4.64 *	14.40
*PSX*	6.47 ± 3.14	6.90	13.20 ± 2.46 *	13.20
*ODX*	8.45 ± 2.38	8.80	24.51 ± 9.46 *	21.10
*OSX*	8.25 ± 3.22	8.60	18.97 ± 5.54 *	19.70
*LDX*	7.21 ± 2.69	7.70	27.81 ± 11.12 *	25.00
*LSX*	7.30 ± 3.41	7.30	21.55 ± 6.91 *	21.00
*TDX*	7.91 ± 2.60	8.00	24.73 ± 10.79 *	22.40
*TSX*	7.92 ± 3.14	7.70	18.78 ± 4.96 *	19.00
*LeDX*	9.79 ± 3.52	9.50	20.39 ± 7.23 *	17.95
*LeSX*	10.45 ± 3.72	10.10	17.68 ± 4.87 *	17.30
*TLDX*	5.11 ± 2.78	4.80	14.97 ± 6.44 *	14.40
*TLSX*	5.29 ± 2.82	5.40	12.79 ± 5.07 *	11.60
*LSG*	4.99 ± 3.34	5.40	25.98 ± 8.83 *	24.30

All values are expressed in °C. (PDX, right pectoral subregion of the sternal region; PSX, left pectoral sub-region of the sternal region; ODX, right homobrachial region; OSX, left homobrachial region; LDX, right lateral view of the thoraco-lumbar region and right dorsal portion of the ventro-lateral region of the abdomen; LSX, left lateral view of the thoraco-lumbar region and left dorsal portion of the ventro-lateral region of the abdomen; TDX, lateral view of the right thigh region; TSX, lateral view of the left thigh region; LeDX, lateral-caudal view of the right leg region; LeSX, lateral-caudal view of the left leg region; TLDX, caudal view of the thigh and right leg region; TLSX, caudal view of the region of the thigh and left leg; LSG, caudal view of the lumbar region, sacral region and dorsal portion of the hip or gluteal region). All values in T1 resulted statistically significant increased compared to T0 (*).

## Data Availability

All data are contained within this article. Interested qualified researchers may request further information by contacting the corresponding author.
